# Application of artificial intelligence in predicting malignancy risk in breast masses on ultrasound

**DOI:** 10.1590/0100-3984.2023.0034

**Published:** 2023

**Authors:** Mariah Carneiro Wanderley, Cândida Maria Alves Soares, Marina Marcondes Moreira Morais, Rachel Malheiros Cruz, Isadora Ribeiro Monteiro Lima, Rubens Chojniak, Almir Galvão Vieira Bitencourt

**Affiliations:** 1 Department of Imaging, A.C.Camargo Cancer Center, São Paulo, SP, Brazil

**Keywords:** Artificial intelligence, Breast neoplasms, Ultrasonography, mammary, Risk assessment., Inteligência artificial, Neoplasias da mama, Ultrassonografia mamária, Medição de risco.

## Abstract

**Objective:**

To evaluate the results obtained with an artificial intelligence-based software for predicting the risk of malignancy in breast masses from ultrasound images.

**Materials and Methods:**

This was a retrospective, single-center study evaluating 555 breast masses submitted to percutaneous biopsy at a cancer referral center. Ultrasonographic findings were classified in accordance with the BI-RADS lexicon. The images were analyzed by using Koios DS Breast software and classified as benign, probably benign, low to intermediate suspicion, high suspicion, or probably malignant. The histological classification was considered the reference standard.

**Results:**

The mean age of the patients was 51 years, and the mean mass size was 16 mm. The radiologist evaluation had a sensitivity and specificity of 99.1% and 34.0%, respectively, compared with 98.2% and 39.0%, respectively, for the software evaluation. The positive predictive value for malignancy for the BI-RADS categories was similar between the radiologist and software evaluations. Two false-negative results were identified in the radiologist evaluation, the masses in question being classified as suspicious by the software, whereas four false-negative results were identified in the software evaluation, the masses in question being classified as suspicious by the radiologist.

**Conclusion:**

In our sample, the performance of artificial intelligence-based software was comparable to that of a radiologist.

## Abstract (pt)

**Objetivo:**

O objetivo deste trabalho foi avaliar os resultados de um *software* baseado em algoritmo de inteligência artificial para predição do risco de malignidade em nódulos mamários.

**Materiais e Métodos:**

Estudo retrospectivo e unicêntrico que avaliou 555 nódulos mamários submetidos a biópsia percutânea em um centro de referência oncológico. Os achados ultrassonográficos foram classificados de acordo com o léxico do BI-RADS. As imagens foram analisadas pelo *software* Koios DS Breast e divididas em benigna ou provavelmente benigna, suspeita baixa ou intermediária, suspeita alta ou provavelmente maligna. O resultado histopatológico foi considerado como padrão ouro.

**Resultados:**

A média de idade das pacientes foi de 51 anos e o tamanho médio dos nódulos foi de 16 mm. A sensibilidade e a especificidade foram de 99,1% e 34,0% para o radiologista e 98,2% e 39,0% para o *software*, respectivamente. O valor preditivo positivo para malignidade para as categorias BIRADS foi semelhante para o radiologista e para o *software*. Foram identificados dois resultados falso-negativos na avaliação pelo radiologista que foram classificados como suspeitos pelo *software*, e quatro resultados falso-negativos na avaliação pelo *software* que foram classificados como suspeitos pelo radiologista.

**Conclusão:**

Na nossa amostra, o *software* de inteligência artificial demonstrou resultados comparáveis à avaliação pelo radiologista.

## INTRODUCTION

Excluding non-melanoma skin cancer, breast cancer is the most common malignant tumor among women worldwide and is the leading cause of cancer death in this population^**(^1^)**^. Imaging is of fundamental importance for the management of patients with breast cancer, especially in the early diagnosis of nonpalpable breast lesions. The imaging modalities most often used in this context are mammography and ultrasound.

Breast ultrasound is a widely used method in Brazil because of its high availability and low cost. It is usually indicated for the complementary evaluation of areas deemed suspicious on mammography or clinical examination, although it can also be used as a screening tool in young patients with dense breasts and a high risk of breast cancer. Albeit equipmentand operator-dependent, ultrasound has been shown to be cost-effective and accurate for the diagnosis of breast lesions^**(^2^)**^.

Despite its high sensitivity for diagnosing breast cancer, conventional ultrasound is known to have relatively low specificity, with a high rate of false-positive results. The literature shows that, for diagnosing breast cancer, the sensitivity of conventional ultrasound ranges from 71.2% to 100.0% and its specificity ranges from 24.0% to 98.8%. For biopsy, the reported rate of a positive result for cancer is only 10-30%. That means that 70-90% of breast biopsies are negative for malignancy, creating unnecessary patient discomfort and anxiety, as well as increasing health care costs^**(^3^,^4^)**^.

Diagnostic imaging is undergoing a paradigm shift, in which the constant incorporation of new technologies has contributed to greater diagnostic accuracy that is adequate to adhere to the current concepts of personalized medicine, with the development of imaging biomarkers that have a direct impact on the management of patients. The incorporation of artificial intelligence (AI) could allow a more accurate, objective, efficient, and reproducible assessment of imaging methods^**(^5^)**^. Studies employing AI have already been applied to different breast imaging modalities and in various clinical settings^**(^6^)**^, including the prediction of breast cancer risk; the detection and classification of lesions; radiogenomics; and the prediction of treatment response and clinical outcomes.

Several authors have used AI algorithms to differentiate between benign and malignant breast masses on breast ultrasound, with promising results^**(^7^-^17^)**^. Although some of these AI-based decision support systems are approved by regulatory agencies, in different countries, there are still no guidelines to recommend the application of AI in ultrasound for clinical practice.

There are as yet no published studies evaluating the application of AI-based software to aid in the classification of breast masses on ultrasound of patients in Brazil. The results of studies carried out abroad, mainly in the United States, might not apply to our reality because of the way in which the examination is carried out in each country. In the United States, the examination is performed by a technician and the images are then evaluated by the physician who will write the report, whereas in Brazil the physician performs the examination, selects the images, and writes the report. Therefore, it is essential to carry out research that evaluates the accuracy of such software when used in Brazil.

The objective of this study was to evaluate the accuracy of AI-based software for predicting the risk of malignancy in breast masses submitted to percutaneous ultrasound-guided biopsy in Brazil.

## MATERIALS AND METHODS

This was a cross-sectional, retrospective, observational single-center study carried out at a cancer referral center. The research project was approved by the institutional review board before the start of data collection, and the requirement for informed consent was waived. We included patients who underwent ultrasound-guided percutaneous biopsy of breast masses between March and December of 2022. Cases for which images were unavailable or inappropriate for analysis were excluded, as were those in which the results of the histological analysis of the biopsy sample were inconclusive or inconsistent with the imaging findings.

The ultrasound images of the cases included in the study were reviewed by five radiologists specializing in breast imaging (one with fewer than five years of experience, two with 5-10 years of experience, and two with more than 10 years of experience), all of whom were blinded to the result of the software evaluation. Ultrasound findings were classified in accordance with the Breast Imaging Reporting and Data System (BI-RADS) lexicon. The images were analyzed with specialized software (Koios DS Breast; Koios Medical, New York, NY, USA), registered in Brazil by the Brazilian Health Regulatory Agency (Reference no. 81464750108). Segmentation of the mass on the image was carried out in two axes in the software, for analysis and prediction of the risk of malignancy. The results were divided into three categories: benign or probably benign (BI-RADS categories 2 and 3, respectively); low or intermediate suspicion (BI-RADS categories 4A and 4B, respectively), and high suspicion or probably malignant (BI-RADS categories 4C and 5, respectively).

The data obtained were stored in a Research Electronic Data Capture (REDCap; Vanderbilt University, Nashville, TN, USA) database for subsequent statistical analysis with the IBM SPSS Statistics software package, version 20.0 (IBM Corp., Armonk, NY, USA). In the descriptive analysis, qualitative variables are presented as absolute and relative frequencies, whereas quantitative variables are presented as main summary measures (mean, standard deviation, and range). To assess the diagnostic validity of the software, the result of the histological analysis of the biopsy sample was considered the reference standard. Sensitivity was calculated as the ratio of true-positive results to the total number of malignant lesions. Specificity was calculated as the ratio of true-negative results to the total number of benign lesions. The positive predictive value was calculated as the ratio of true-positive results to the total number of positive results, and the negative predictive value was calculated as the ratio of true-negative results to the total number of negative results. Accuracy was calculated as the ratio of the sum of true-positive and true-negative results to the total number of lesions evaluated.

## RESULTS

A total of 555 breast masses, in 509 patients, were included; 22 cases were excluded. The mean age of the patients was 51.0 ± 15.3 years (range, 16-90 years), and the mean mass size was 16.0 ± 11.6 mm (range, 3-114 mm). The characteristics of the masses on ultrasound are described in Table 1.

**Table 1:** Ultrasound characteristics of the masses included in the study (n = 555).

Variable	Category		n (%)	
Shape	Oval		251 (45.2)	
	Round		29 (5.2)	
	Irregular		275 (49.5)	
Margins	Circumscribed		200 (36.0)	
	Non-circumscribed		355 (64.0)	
Orientation	Parallel		390 (70.3)	
	Not parallel		165 (29.7)	
Echo pattern	Hypoechoic		430 (77.5)	
	Isoechoic		28 (29.7)	
	Hyperechoic		7 (1.3)	
	Heterogeneous		66 (11.9)	
Posterior features	None		447 (80.5)	
	Shadowing		68 (12.3)	
	Enhancement		38 (6.8)	
	Combined pattern		2 (0.4)	
Size	0-10 mm		196 (35.3)	
	11-20 mm		311 (56.0)	
	> 20 mm		48 (8.7)	
BI-RADS	2a		14 (2.5)	
	3		102 (18.4)	
	4A		181 (32.6)	
	4B		74 (13.3)	
	4C		135 (24.3)	
	5		49 (8.8)	

The histological diagnosis was obtained in a core biopsy sample in 466 cases (84.0%) and in a vacuum-assisted biopsy sample in 89 (16.0%). In the histological analysis, 333 lesions (60.0%) were classified as benign and 222 (40.0%) were classified as malignant.

The sensitivity, specificity, positive predictive value, negative predictive value, and accuracy were 99.1%, 34.2%, 50.1%, 98.3%, and 60.2%, respectively, for the radiologist evaluation, compared with 98.2%, 39.0%, 51.8%, 97.0%, and 62.7%, respectively, for the software evaluation (Table 2).

**Table 2:** The BI-RADS classifications assigned by the radiologist and by the AI-based software, in comparison with the histological classification (reference standard).

BI-RADS classification	Histological classification			
	Benign n (%)	Malignant n (%)		Total n (%)
Radiologist evaluation				
Category 2-3	114 (98.3)	2 (1.7)		116 (100.0)
Category 4A-4B	205 (80.4)	50 (19.6)		255 (100.0)
Category 4C-5	14 (7.6)	170 (92.4)		184 (100.0)
Software evaluation				
Category 2-3	130 (97.0)	4 (3.0)		134 (100.0)
Category 4A-4B	193 (69.7)	84 (30.3)		277 (100.0)
Category 4C-5	10 (6.9)	134 (93.1)		144 (100.0)

**Figure 1:**
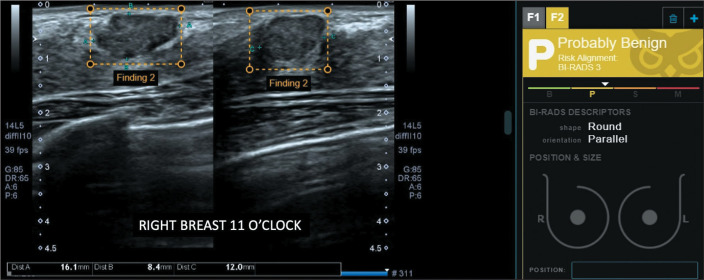
Non-circumscribed mass in the right breast, classified by the radiologist as BI-RADS 4A (low suspicion) and by the software as BI-RADS 3 (probably benign). The histopathological diagnosis was fibroadenoma.

We identified two false-negative results in the radiologist evaluation that were classified as suspicious by the software and four false-negative results in the software evaluation that were classified as suspicious by the radiologist. All lesions classified as benign (BI-RADS 2), probably benign (BI-RADS 3), or of low suspicion (BI-RADS 4A) by the radiologist and as benign (BI-RADS 2) or probably benign (BI-RADS 3) by the software (n = 117) were classified as benign in the histological analysis of the biopsy sample (Table 3). Figures 1 through 4 illustrate examples of cases evaluated in the study.

**Table 3:** Comparison between the BI-RADS classifications assigned by the radiologist and those assigned by the software, in relation to the histological classification (reference standard).

BI-RADS classification		Histological classification		Total n (%)
		Benign n (%)	Malignant n (%)	
Radiologist	Software			
evaluation	evaluation			
Category 3	Category 2-3	66 (100.0)	0 (0.0)	66 (100.0)
	Category 4A-4B	32 (97.0)	1 (3.0)	33 (100.0)
	Category 4C-5	2 (66.7)	1 (33.3)	3 (100.0)
Category 4A	Category 2-3	54 (100.0)	0 (0.0)	54 (100.0)
	Category 4A-4B	112 (89.6)	13 (10.4)	125 (100.0)
	Category 4C-5	0 (0.0)	2 (100.0)	2 (100.0)
Category 4B	Category 2-3	2 (40.0)	3 (60.0)	5 (100.0)
	Category 4A-4B	32 (55.2)	26 (44.8)	58 (100.0)
	Category 4C-5	5 (45.5)	6 (54.5)	11 (100.0)
Category 4C	Category 2-3	1 (100.0)	0 (0.0)	1 (100.0)
	Category 4A-4B	10 (22.7)	34 (77.3)	44 (100.0)
	Category 4C-5	3 (3.3)	121 (89.6)	124 (100.0)
Category 5	Category 2-3	0 (0.0)	1 (100.0)	1 (100.0)
	Category 4A-4B	0 (0.0)	10 (100.0)	10 (100.0)
	Category 4C-5	0 (0.0)	38 (100.0)	38 (100.0)

**Figure 4:**
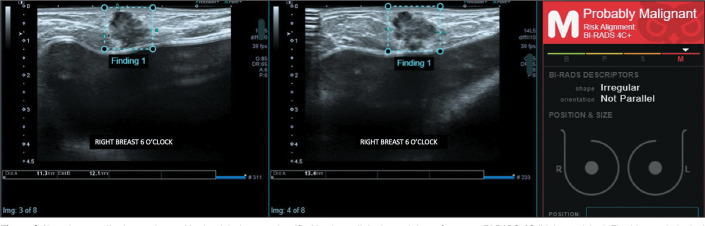
Non-circumscribed mass located in the right breast, classified by the radiologist and the software as BI-RADS 4C (high suspicion). The histopathological diagnosis was luminal B invasive breast carcinoma of no special type.

## DISCUSSION

The results of the present study demonstrate that, for predicting the risk of malignancy in breast masses submitted to ultrasound-guided percutaneous biopsy, the sensitivity, specificity, positive predictive value, negative predictive value, and diagnostic accuracy of the AI-based software were similar to those of radiologists at a cancer referral center in Brazil. In addition, all lesions that classified as benign, probably benign, or of low suspicion by the radiologist and were classified as benign or probably benign by the software were categorized as benign in the histological analysis, demonstrating the potential of this tool to reduce the number of unnecessary biopsies.

The software used in the present study has been approved by the U.S. Food and Drug Administration and is routinely used at referral centers worldwide. Mango et al.^**(^13^)**^ evaluated the performance of this tool with multiple radiologists and found that, when the evaluation of the support tool was combined with that of a radiologist, the accuracy of the ultrasound evaluation of breast lesions was better than was that of the radiologist evaluation alone. The authors also observed significant lesion downgrading (from BI-RADS 4A to BI-RADS 3), as well as less interobserver and intraobserver variability, which is critical for standardizing the assessment and reducing the number of unnecessary biopsies. Similar to what was observed in the present study, Browne et al.^**(^17^)**^ demonstrated that most biopsies of lesions classified as BI-RADS 3 could be avoided using the same tool. Studies using other AI-based tools have obtained similar results^**(^11^,^16^)**^.

On the basis of the findings of the present study and of the previously cited studies, we believe the following: that clinical data and the comparison with previous examinations should always be taken into account for the indication of biopsy in breast masses, regardless of the evaluation made by the software; that biopsy can be safely avoided in lesions classified as BI-RADS 2 or 3 by the radiologist and the software; that masses classified as BI-RADS 4A by the radiologist could be downgraded to BI-RADS 3 when they are classified as BI-RADS 2 or 3 by the software; and that masses classified as BI-RADS 4B, 4C, or 5 by the radiologist should always be submitted to biopsy, regardless of the software evaluation, which can, however, be useful for an adequate radiological-pathological correlation. In our study sample, following those guidelines could have avoided a biopsy in 117 (21.1%) of the cases, without missing any malignant lesions, and 54 (29.8%) of the 181 lesions initially classified as BI-RADS 4A could have been reclassified as BI-RADS 3.

This study has the limitations inherent to a retrospective study. Some cases were excluded from the analysis because the images on file were not appropriate for analysis, including cases in which the lesions were documented on only one axis or only on Doppler images. Lesions classified as benign or probably benign were biopsied at the discretion of the attending physician, probably on the basis of other clinical data. In addition, only masses were included in the study, because the software has not yet been trained to evaluate non-mass lesions on ultrasound. Because the study was conducted at a referral center, the radiologists who performed the ultrasound examination had more experience in performing breast ultrasound than would those working at less specialized centers, and that difference could have influenced the results obtained.

In conclusion, in our sample, the AI-based software tested demonstrated results comparable to the evaluations made by radiologists at a referral center. This tool can be useful in predicting the risk of malignancy in breast masses identified on ultrasound, especially at facilities with less experience in breast ultrasound, making the indication for percutaneous biopsies more accurate.

## Figures and Tables

**Figure 2:**
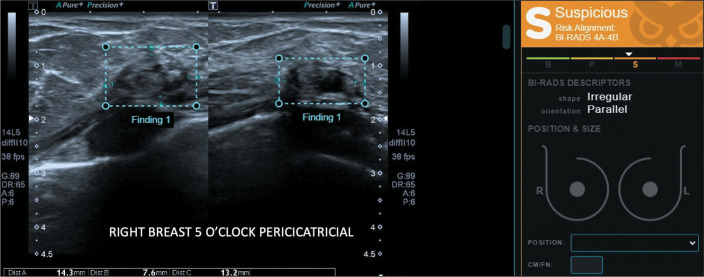
Steatonecrosis related to the site of previous surgical manipulation, confirmed by biopsy and stable in follow-up examinations, classified by the software as a BI-RADS 4A-4B (lowto intermediate-suspicion) mass.

**Figure 3:**
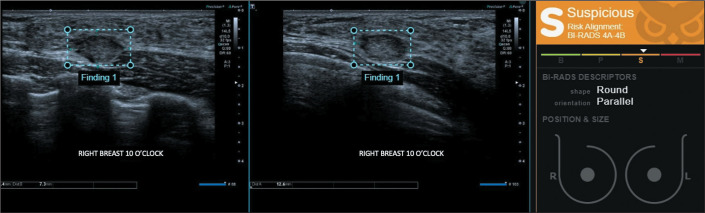
A mass in the right breast, classified by the radiologist as BI-RADS 3 (probably benign) and by the software as BI-RADS 4A-4B (low to intermediate suspicion). Percutaneous ultrasound-guided biopsy was performed, and the histopathological diagnosis was triple-negative invasive breast carcinoma of no special type.
